# Demonstrative Midwifery

**Published:** 1850-05

**Authors:** 


*Demonstrative Midwifery—Differences among Physicians.*—The following letter has been handed to us, with the request that it be inserted in this Journal :—

LOCKPORT, April 2, 1850

DR. FLINT:—*Sir*,—The undersigned have read the editorial articles, the correspondence, and resolutions of the class, and the letter of the seventeen medical gentlemen of the city of Buffalo, contained in the February and March numbers of the Buffalo Medical Journal, on the subject of Demonstative Midwifery; and have also conversed with a number of the class who attended the Demonstration, and are happy to say, that the plan commends itself to their most cordial approbation.

CALEB HILL, M. D.,
DANIEL MORSE, M. D.,
J. S. SHULER, M. D.,
B. L. DELANO, M. D.,
WM. M'COLLUM, M. D.,
D. S. FASSETT, M. D.,
W. B. GOULD, M. D.,
JAMES C. MAY, M. D.,
J. K. SKINNER, M. D.

In a letter accompanying the above, it is stated, that "all the faculty of this place, (Lockport) save one (and he was absent), have cheerfully put their names to the enclosed paper." We do not propose, at that this time, to enlarge on the subject of the letter, although we have no reluctance to discuss it, in any, or all of its bearings, as a proposed improvement in the system of teaching preparatory for the practical duties of the accoucheur. It is, we trust, needless to say, that our columns are open to any who desire to discuss it, in a proper spirit, whether the object be to sustain or oppose it. As our readers are aware, the subject has elicited an expressed difference of opinion by several medical gentlemen of this place, associatted for that purpose, and this it is which has called out the views of the physicians of Lockport, who appear to regard it with more unanimity of sentiment, than do the medical fraternity of Buffalo. It is not a new saying that Doctors disagree. It would certainly be unjust to refuse to the medical profession the right to differ—a privilege conceded to every other class. No one can complain of differences of opinion, honestly cherished, be they ever so stoutly maintained. Propriety, not less than good policy, however, manifestly requires that discussions growing out of such differences should be restricted, as far as practicable, within the pale of the profession. "*Who shall decide* when doctors disagree?" Most assuredly not the public. The effort to involve popular *sentiment*, or the prejudices of a *community* in professional disputes, always evinces a a sinister purpose, is in direct violation of medical ethics, and tends, more than any thing else, to lower the profession in popular estimation. On this point the code adopted by the National Medical Association, and by local associations generally throughout the country, is explicit, and as the topic is one concerning which all should have clear views, we quote from the instrument referred to, as follows:—

*Of differences between Physicians.*—Diversity of opinion, and opposition of interest, may, in the medical, as in other professions, sometimes occasion controversy and even contention. Whenever such cases unfortunately occur, and cannot immediately be terminated, they should be referred to the arbitration of a sufficient number of physicians, or a *court-medical.*

As peculiar reserve must be maintained by physicians towards the public, in regard to professional matters, and as there exist numerous points in medical ethics and etiquette through which the feelings of medical men may be painfully assailed in their intercourse with each other, and which cannot be understood or appreciated by general society, neither the subject matter of such differences nor the adjudication of arbitrators should be made public, as publicity in a case of this nature may be personally injurious to the individuals concerned, and can hardly fail to bring discredit on the faculty.

To return to the subject of *demonstrative midwifery*, so far as expressions of opinion have come to our knowledge, they go to sustain the belief hazarded by us in the February number of this Journal, that the "plan must

